# Biosensors for Deoxynivalenol and Zearalenone Determination in Feed Quality Control

**DOI:** 10.3390/toxins13070499

**Published:** 2021-07-17

**Authors:** Krisztina Majer-Baranyi, Nóra Adányi, András Székács

**Affiliations:** 1 Food Science Research Group, Institute of Food Science and Technology, Hungarian University of Agriculture and Life Sciences, Herman Ottó út 15, H-1022 Budapest, Hungary; adanyine.kisbocskoi.nora@uni-mate.hu; 2 Agro-Environmental Research Centre, Institute of Environmental Sciences, Hungarian University of Agriculture and Life Sciences, Herman Ottó út 15, H-1022 Budapest, Hungary; szekacs.andras@uni-mate.hu

**Keywords:** biosensors, zearalenone, deoxynivalenol, immunosensors, feed, antibody, aptamer, molecularly imprinted polymer

## Abstract

Mycotoxin contamination of cereals used for feed can cause intoxication, especially in farm animals; therefore, efficient analytical tools for the qualitative and quantitative analysis of toxic fungal metabolites in feed are required. Current trends in food/feed analysis are focusing on the application of biosensor technologies that offer fast and highly selective and sensitive detection with minimal sample treatment and reagents required. The article presents an overview of the recent progress of the development of biosensors for deoxynivalenol and zearalenone determination in cereals and feed. Novel biosensitive materials and highly sensitive detection methods applied for the sensors and the application of these sensors to food/feed products, the limit, and the time of detection are discussed.

## 1. Introduction

Mycotoxin contamination is one of the most important problems in food and feed safety. According to previous studies, 25–50% of crops harvested worldwide are contaminated with different types of mycotoxins [1]. *Fusarium* species are the most widespread pathogens in cereals, and *Fusarium* toxins are the most reported mycotoxins in raw agricultural commodities [2]. Therefore, mycotoxins produced by *Fusarium* moulds significantly affect feed quality and safety and also represent a prominent issue in feed quality control after the most hazardous contaminants aflatoxins of *Aspergillus* origin. Accordingly, as most alerts in official food and feed monitoring mostly refer to aflatoxin contamination [3], most monitoring activities and analytical method development efforts are geared towards aflatoxins. Nonetheless, growing attention is paid to *Fusarium* mycotoxins as well, partly due to their spread caused by climate change and partly due to their well-known toxicological significance. Among *Fusarium* mycotoxins, deoxynivalenol (DON) and zearalenone (ZON), as well as their metabolites 3- and 15-acetyl-DON, α-, and β-zearalenol, are of special importance as they are formed under field conditions prior to harvest, being highly stable during storage and difficult to degrade by thermal processing [4,5,6]. Especially wheat, barley, oats, rye, corn, and triticale are vulnerable to *Fusarium* infection, and compared to other cereals, they are also frequently contaminated mostly with DON and ZON [7]. Low-level contamination of *Fusarium* toxins is very frequent. DON and ZON are typically found in more than 50% and about 80%, respectively, of food samples tested in studies conducted between 2010 and 2015 in the EU [8]. DON, also known as vomitoxin, is of primary concern due to its genotoxicity, but it can also cause slow growth, lowered milk production in cattle, feed refusal, reduced egg production in laying hens, intestinal haemorrhage, and suppression of immune responses. ZON is problematic due to its hormonal effects causing changes in the reproductive system and reduced fertility. The use of toxin-contaminated feeds in livestock farming can cause a variety of adverse health effects in farm animals and a corresponding high degree of economic loss. Furthermore, contaminated feed can pose a health risk to humans indirectly, while mycotoxin carry-over is possible to milk, meat, and eggs; therefore, systematic control of mycotoxin content in feeds is of great importance. Although ZON, DON, and their metabolites are not of major concern due to their occurrence in milk, their presence has been reported in several studies. In an Italian study, 185 cow’s milk-based infant formula products were investigated for ZON and its metabolites. ZON, α-, and β-zearalenol were detected in 9%, 26%, and 28.6% of the samples, respectively, with a maximum level of the latter metabolite of 73.2 ng/mL [9]. A technical survey from New Zealand reported that 0.06–0.08% of ZON residues mainly in form of α- and β-zearalenol can be secreted into milk, while DON residues occur in milk mainly in form of its diepoxy derivative exerting lower toxicity than the parent mycotoxin [10]. The EU has established maximum permitted levels and guidance levels of certain mycotoxins in feed, which should be routinely monitored. The guidance levels for ZON is 100–500 µg/kg in complementary and complete feeding-stuffs and 2–3 mg/kg for feed material, and for DON, it is 900 μg/kg in complementary and complete feeding-stuffs, 8 mg/kg in cereals and cereal products, and 12 mg/kg in maize by-products [11]. Commonly used techniques, such as high-performance liquid chromatography (HPLC) hyphenated with different detectors [12,13,14], liquid chromatography coupled with mass spectrometry (LC-MS) [15], liquid chromatography-tandem mass spectrometry (LC-MS/MS) [16,17,18], and gas chromatography-tandem mass spectrometry (GC-MS/MS) [19,20], for mycotoxin determination in food and feed have been powerful tools, as they provide proper sensitivity and accuracy in quantitative determination, but they are time-consuming, laborious, expensive, and require advanced instrumentation and trained staff [21]. In contrast, technically simple thin-layer chromatography (TLC) is also an excellent tool for rapid routine testing [22,23]; however, its sensitivity is unsatisfactory because of the even stricter EU limits. It is therefore essential to develop analytical methods that can detect the target analytes with sufficient sensitivity and accuracy and at the same time are inexpensive, fast, rely on simple measurement techniques, and allow on-site applications. The development and use of biosensors in food and feed analysis may efficiently address this challenge. This paper aims to provide an overview of recent advances and current trends in biosensor development for ZON and DON determination.

## 2. The Use of Sensorics for Determination of DON and ZON

Biosensors can be defined as a device incorporating an active biological sensing element (an enzyme, a tissue, living cells, antibodies, molecularly imprinted polymers (MIP), aptamers, DNA/RNA) connected to a transducer that converts the observed physical or chemical changes into a measurable signal. Biosensors can be classified according to the applied recognition elements (enzyme sensors, immunosensors, aptasensors, etc.) and also according to the signal transduction method: optical, electrochemical, piezoelectric, and thermometric; however, the latter application is not common in food and feed analysis. For mycotoxin determination, immunosensors are the most commonly applied analytical tools among biosensors, but beside that, MIP-based sensors and aptasensors (as artificial recognition element-based sensors) are also emerging techniques. Immunosensors employ antibodies, antibody fragments, antigens, or antigen conjugates as biomolecular recognition elements, and the specific antigen-antibody binding event is detected and converted to a measurable signal by the transducer. The basic working principle of the immunosensor set-up is that the specific binding of the antibody or antigen immobilised on the transducer to the antigen or antibody in the sample produces an analytical signal that varies dynamically with the concentration of the analyte of interest. The formation of the immunocomplex can be determined either by label-free methods by directly measuring the physical changes induced by the binding event or by label-based modes using detection specific labels. For mycotoxin determination, both labelled and label-free immunosensors incorporated with various types of transducers are extensively researched and developed.

### 2.1. Optical Immunosensors

Nowadays, beside electrochemical immunosensors, the use of optical transducers has taken the lead in immunosensor development for mycotoxin determination because optical detection allows the construction of sensitive, simple, inexpensive, and portable analytical devices for on-site monitoring and also enables direct, real-time detection of various analytes. Optical biosensing can be divided into two general modes: label-free and label-based mode. Label-free biosensors do not require the use of any label to monitor the biorecognition event, while label-based protocols use specific labels like fluorescent dyes, enzymes, or nanoparticles, and the optical signal is generated by colorimetric, fluorescent, or luminescent methods [24,25]. Although these label-based methods are very sensitive and widely used, the performance of the sensor depends on the efficiency of the labelling step. Thus, the use of label-free biosensors may be preferable to the use of label-based ones, as they offer simple, rapid measuring procedures and enable real-time monitoring of the binding reaction. Of course, label-free optical biosensors also have disadvantages, especially in the determination of small molecules such as mycotoxins, as the sensor response often depends on the size of the analyte, and these analytes are mostly not chromogenic or fluorescent [26].

#### 2.1.1. Label-Free Optical Immunosensors

**Figure 1:**
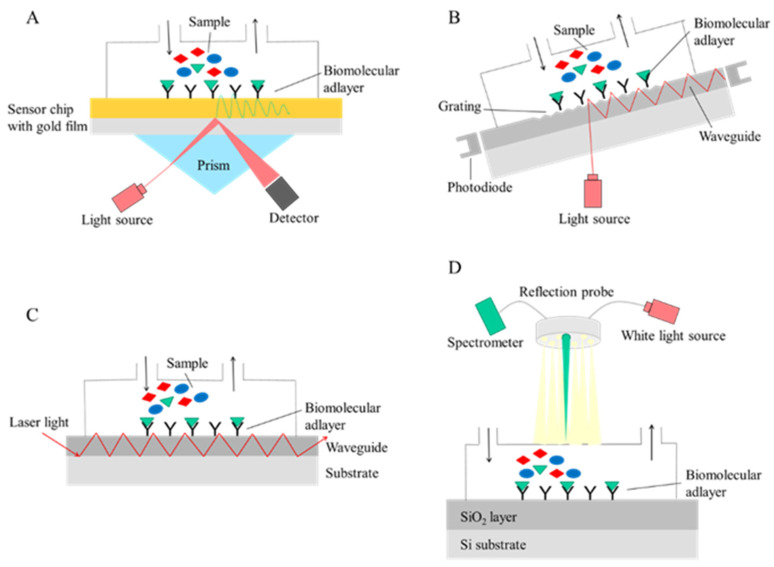
Operating principles of label-free optical immunosensors. (**A**) Surface plasmon resonance (SPR); (**B**) optical waveguide lightmode spectroscopy (OWLS); (**C**) planar waveguide; (**D**) white light reflectance spectroscopy.

Surface plasmon resonance (SPR) technique has gained great attention in biosensor development lately. The technique was introduced in the early 1990s and since then become a powerful analytical tool in the risk assessment of contaminants in food and feed [27]. The SPR phenomenon occurs at the gold surface of the sensor chip when an incident polarised laser light beam strikes the surface at a particular angle through a prism (Figure 1A). It generates electron charge density waves called plasmons, which cause intensity reduction of the reflected light at this angle [28]. In the SPR immunosensor, immunogens (antibody or antigen) are immobilised on the gold layer of the chip mounted on a glass support. The binding of the analyte to the sensor surface causes a local change in refractive index, and corresponding shifts of the coupling angle are monitored in real time. SPR-based biosensors have received considerable attention in the past decades as they allow fast, reliable, and label-free detection of analytes [29]. In addition, they are suitable for real-time monitoring of the interaction kinetics; moreover, the biosensor chips are reusable. Another advantage of the SPR technique is that several measurements can be performed in parallel on a single sensor using multi-channel measurement. As several mycotoxins may be present simultaneously in feed or food samples, multiplex analysis is particularly relevant. Despite the fact that the SPR technique in biosensor research is being studied very extensively [30,31,32,33], only a few sensor development efforts suitable for ZON or DON determination have been investigated in recent years.

Recently, Wei et al. [34] reported an SPR-based biosensor for the simultaneous determination of aflatoxin B1 (AFB1), ochratoxin A (OTA), ZON, and DON in corn and wheat. The limit of detection (LOD) for AFB1, OTA, ZON, and DON were identified as 0.59 ng/mL, 1.27 ng/mL, 7.07 ng/mL, and 3.26 ng/mL, respectively. Average recoveries were between 85% and 115%. Joshi et al. [35] developed two types of SPR-based biosensors for the detection of mycotoxins in barley. First, a double 3-plex assay was developed for the detection of DON, ZON, and T-2 toxin on the first chip and for OTA, fumonisin B1 (FB1), and AFB1 on the second chip using SPR. After determining the optimal conditions, the assay was transferred to a 6-plex format (six different mycotoxins determined on a single chip) in a portable nanostructured imaging surface plasmon resonance (iSPR) instrument, and the two assays were compared. The advances of iSPR technique over conventional SPR are the visualisation of the entire sensor surface in real time to monitor hundreds of molecular interactions simultaneously, and also multiplex detection is available. Results showed that DON, T-2, ZON, and FB1 could be detected at sufficient levels in barley samples according to the EC guidelines, but for OTA and AFB1, sensitivities should be improved when SPR was used for determination. The portable 6-plex iSPR was less sensitive but still allowed detection of DON, T-2, ZON, and FB1 at relevant levels. The sensitivities (IC_50_ values) obtained by iSPR biosensor in an assay buffer for T-2, FB1, and ZON were 10 ng/mL, 8 ng/mL, and 25 ng/mL, respectively.

A rapid and sensitive iSPR assay was developed for *Fusarium* toxins by Hossain and Maragos [36] using secondary antibody with gold nanoparticles (AuNPs) as an amplification tag to determine DON, ZON, and T-2 toxin in wheat. LODs were 15 µg/kg for DON, 24 µg/kg for ZON, and 12 µg/kg for T-2 toxin. Sensor chips could be reused for over 46 cycles without significant signal loss, and it took 17.5 min to measure a sample, including the regeneration steps. The same research group developed an iSPR-based immunosensor for T-2 and T-2 toxin 3-glucoside (T2-G), so-called “masked” mycotoxin, determination in wheat, which is a niche in the field of research [37]. In their experiment on a carboxyl functionalised sensor surface, T-2-protein conjugate was immobilised using 1-ethyl-3-(3-dimethylaminopropyl) carbodiimide with N-hydroxysuccinimide (EDC-NHS) method. A competitive immunoassay format was applied to detect the mycotoxins, and a secondary antibody labelled with AuNPs was used for signal amplification. The LOD was 48 µg/kg of T-2 and 36 µg/kg of T-2-G; the recoveries ranged between 86–90%. Hu et al. [38] could achieve LODs for AFB1, OTA, and ZON as low as 8, 30, and 15 pg/mL, respectively, with their iSPR immunosensor using AuNPs for signal amplification.

Another emerging technique in the field of optical immunosensor development is the optical waveguide lightmode spectroscopy (OWLS) technique that enables monitoring molecular interactions on the sensor surface in a label-free manner in real-time (Figure 1B). The basic principle of the OWLS method is that linearly polarised He-Ne laser light is coupled by a diffraction grating into the waveguide layer. The incoupling is a resonance phenomenon that occurs at a defined angle of incidence that depends on the refractive index of the medium covering the surface of the waveguide. In the waveguide layer, light is guided by total internal reflection to the edges, where it is detected by photodiodes. By varying the angle of incidence of the light, the mode spectrum can be obtained from which effective refractive indices are calculated for both the electric and magnetic modes. The sensor consists of a glass substrate with a lower refractive index and a thin (160–220 nm) waveguide layer with a higher refractive index mounted on the top in which a fine optical grating (2400–3600 line/mm) is formed for in- or outcoupling of the light [39].

For DON measurement, Majer-Baranyi et al. [40] presented a label-free OWLS-based immunosensor. In their research, the sensor was modified by 3-aminopropyltriethoxysilane (APTS), and a DON-ovalbumin conjugate was immobilised via glutaraldehyde (GA). With the optimised sensor, DON content of spiked wheat flour samples was investigated using a competitive assay method where DON was quantitatively detectable in the 0.005–50 mg/kg concentration range, and it took 10 min to measure a sample, offering fast and sensitive determination of DON. Székács et al. [41] developed a competitive OWLS-based immunosensor for ZON determination in maize samples. In the competitive assay method, a ZON-bovine serum albumin (BSA) conjugate was immobilised on the sensor surface using three different surface modification methods. According to their results, the epoxy-modified sensors provided lower binding efficacy and reproducibility; when using amino-silanised sensor chips for immobilisation either by GA (APTS/GA) or succinic anhydride (SA) and EDC-NHS (APTS/SA/EDC-NHS) the detection range of ZON were the same in both cases, but for further application, the APTS/SA/EDC-NHS sensor was chosen due to the better reproducibility and longer shelf-life. The LOD of ZON was 0.002 pg/mL, and the dynamic measuring range was between 0.01 and 1 pg/mL.

Recently, another waveguide-based immunosensor for ZON detection was published also using a planar waveguide (PW) for the sensor set-up [42] (Figure 1C). The working principle of the sensor is as follows: circularly polarised laser light is incoupled into the planar waveguide, which propagates through by multiple internal reflections, and the outcoming light is collected by a charge-coupled device (CCD) array photodetector. The sensing principle is based on the different behaviour of the s- and p-components of polarised light. Changes in the refractive index of the covering media cause phase shifts between p- and s-polarisations of light, which are converted to a multiperiodic signal by a polariser and detected by a CCD photodetector. For ZON determination, polyclonal ZON-specific antibodies were immobilised on the functionalised surface, and the binding of ZON was detected in a direct manner. The LOD of the method was 0.01 ng/mL, and the dynamic working range was between 0.01–1000 ng/mL.

Another emerging label-free optical sensor technique is white light reflectance spectroscopy (WLRS), where a broadband light from a light source is emitted and guided vertically to the surface by a reflection probe consisting of six fibres distributed on the periphery of the circle-shaped probe, while the reflected light from the sample is collected by the optical fibre positioned in the centre of the probe and directed to the spectrometer (Figure 1D). The sensor consists of two layers: a Si substrate and, on top of this, a thicker silicon dioxide layer where the biomolecules can be immobilised. The emitted white light is reflected from the sensor consisting of layers with different refractive indexes, resulting in an interference spectrum that is recorded by the spectrometer. Due to biomolecular interactions on the surface, the spectra shift to higher wavelengths [43]. A fast WLRS-based immunosensor for DON determination in wheat and maize samples was reported by Anastasiadis et al. [44], where DON-ovalbumin conjugate was immobilised on the aminosilanised sensor surface. A competitive immunoassay was performed where DON presented in the sample and DON immobilised on the sensor surface were competed for the anti-DON monoclonal antibody binding sites. The primary immunoreaction was followed by a signal enhancement step using an anti-mouse IgG secondary antibody. With the optimised sensor, wheat and maize samples were investigated. In the spiked grain samples, the LOD of DON was 62.5 μg/kg in both cases, while the linear response range was broadened up to 12.5 mg/kg. The measurement was completed within 17 min, including regeneration step, and a single chip could be reused 20 times.

The statistical parameters of the measurements, the cross reactivity, and the matrix analysed of optical immunosensors for DON and ZON detection are summarized in Table 1.

#### 2.1.2. Label-Based Optical Immunosensors

**Figure 2:**
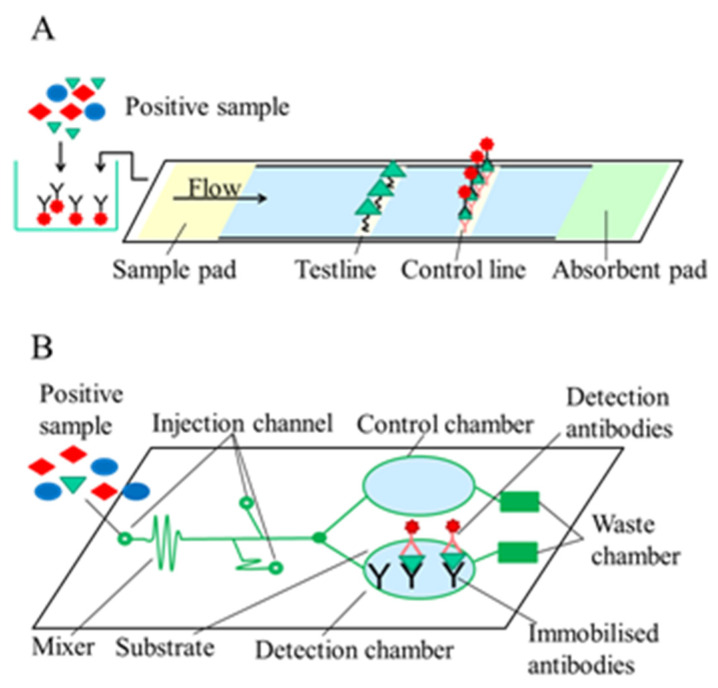
Operating principles of label-based optical immunosensors. (**A**) Paper-based microfluidic device; (**B**) microfluidic capillary chip.

Jiang et al. [45] presented a paper-based microfluidic device (DON-Chip) for DON determination. In the competitive immunoassay, AuNPs were used for labelling. For signal reading, a low-powered digital microscope connecting to a computer’s USB port was used for image acquisition and signal analysis to enable on-site determination. Detection of DON in aqueous extracts of food and feed was carried out by DON-chip, and the results were compared by those obtained by commercial DON ELISA, which showed linear correlation. The LOD of DON was 4.7 ng/g, and the linear working range was between 0.01–20 μg/g. For simultaneous determination of ZON and DON, Jin et al. [46] developed a novel dual near-infrared fluorescence-based lateral flow immunosensor (NIR-based LFIA). On the nitrocellulose membrane, DON and ZON conjugated to BSA were immobilised in the same test line. The anti-ZON and anti-DON antibodies were labelled by near-infrared dyes with distinct fluorescence characteristics as detection reagents. With the optimised sensor, the ZON and DON content of maize samples were determined with a LOD of 0.55 μg/kg and 3.8 μg/kg, respectively. The assay took 20 min to perform, providing a fast and sensitive tool for simultaneous determination of two mycotoxins (Figure 2). 

A multiplexed microfluidic capillary chip with smartphone detection for DON, OTA, and AFB1 determination was demonstrated [47]. A competitive immunoassay format was used to detect mycotoxins simultaneously, where mycotoxin-BSA conjugates were immobilised on a polydimethylsiloxane (PDMS) surface. Toxins present in the sample compete with the toxins immobilised on the surface for the binding site of the polyclonal antibodies conjugated with horseradish peroxidase. After that, hydrogen peroxide as a substrate and tetramethylbenzidine (TMB) as a chromophore were added, and the colorimetric signal was detected by a smartphone and analysed in ImageJ software. The assay could be performed in less than 10 min with a LOD of 10 ng/mL for DON, making the assay capable of fast, on-site analysis. Another smartphone-based sensor was developed by Liu et al. [48] using a dual fluorescence or colour detection mode device integrated with two lateral flow immunoassays for multiplex mycotoxin (DON, ZON) determination in cereals. When fluorescence detection was applied, the assays were more sensitive, but recoveries from maize for both formats were the same.

### 2.2. Electrochemical Immunosensors

**Figure 3:**
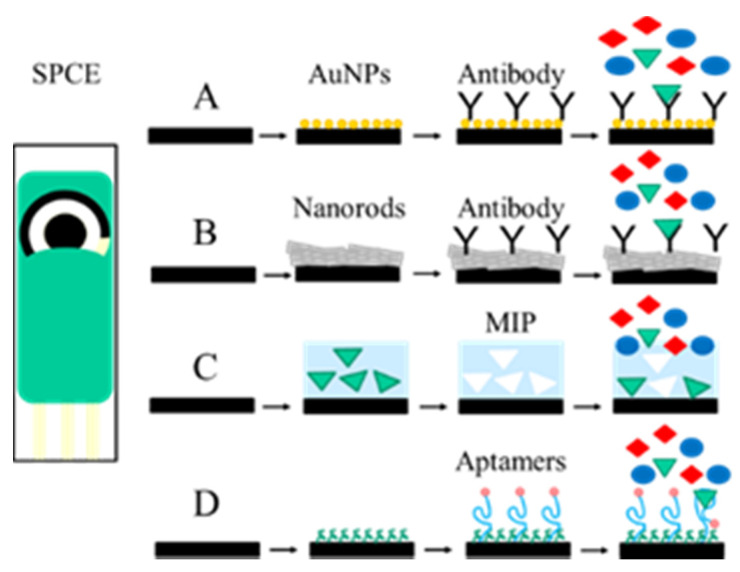
Structure principles of electrochemical sensors. (**A**) Gold nanoparticles (AuNPs); (**B**) nanorods, nanotubes (Au, C, etc.); (**C**) molecular imprinting polymers (MIP); (**D**) aptamers.

In the electrochemical biosensors, the reaction between the target molecule and the recognition element by using electrochemical dyes or enzymatic reactions generates changes in the signal for conductance or impedance, measurable current, or change accumulation, which can be quantified by voltammetric, potentiometric, amperometric, or conductometric techniques [49] (Figure 3). The use of electrochemical biosensors is very common due to their high sensitivity, selectivity, low cost, simplicity, and in some cases their miniaturisation, portability, and integration into automated devices [50,51,52]. In the last decade, the use of screen-printed electrodes (SPE) in electrochemical biosensor development has received great attention because they can be made of different materials and shapes and can be modified with a wide variety of nanomaterials, such as carbon nanotubes, graphene, and metallic nanoparticles as gold, silver, and magnetic nanoparticles coupled with different biological recognition elements (DNA, RNA, aptamers, enzymes, antibodies) [53,54,55,56,57,58,59] (Figure 3A,B).

An electrochemical immunosensor to determine ZON in maize using modified screen-printed carbon electrodes (SPCE) was developed by Riberi et al. [60]. On the surface of the SPCE modified with multi-walled carbon nanotubes/polyethyleneimine dispersions and AuNPs, ZON polyclonal antibodies were immobilised. A competitive immunoassay was used for ZON determination where ZON presented in the sample, and a horseradish peroxidase (HRP)-labelled ZON conjugate competed for the limited amount of polyclonal antibodies immobilised on the surface. After that, hydrogen peroxide was added, and a steady-state current was obtained, which was proportional to the amount of ZON in the samples and was detected at a potential of −0.3V by amperometry. The biosensors showed good stability during at least four days. The calibration curve was linear in the ZON concentration range from 0.1 to 100 pg/mL.

A differential pulse voltammetry (DPV) detection-based immunosensor using disposable SPE was prepared for ZON determination by Goud et al. [61]. On the activated sensor surface, a ZON-BSA conjugate was immobilised by the EDC/NHS method. A competitive assay format was used for ZON determination, and alkaline phosphatase-labelled antibody and 1-naphthyl phosphate (1-NP) as a substrate was used to detect primary antibody binding to the surface. The produced 1-naphthol was detected via DPV, which allowed the determination of the ZON concentration of the sample. The LOD was 0.25 ng/mL, and the dynamic measuring range of ZON was 0.25–256 ng/mL.

A mesoporous silica-modified SPCE-based immunosensor was presented by Regiart et al. [62]. For the immunosensor anti-ZON antibodies were immobilised by GA on the surface of the modified electrode. During measurement, ZON presented in the sample was recognised and bound to the immobilised antibodies on the surface of the electrode. Then, to detect immunocomplex formation, HRP-conjugated anti-ZON antibodies were added, and hydrogen peroxide with 4-tert-butylcatechol (4-TBC) were used in a substrate and chromophore solution. The HRP enzyme catalyzes the oxidation of 4-TBC to 4-tert-butylbenzoquinone. The enzymatic product was detected by amperometry at −100 mV. The measured current was proportional to the concentration of ZON present in the sample. The linear measuring range of ZON detection was 1.88–45 ng/mL, and the LOD was 0.57 ng/mL in *Amaranthus cruentus* seeds.

An electrochemical immunosensor fabricated on indium tin oxide (ITO)-coated glass was introduced by Lu et al. [63] for multiple mycotoxin determination. A dual-channel three-electrode sensor consisted of two working electrodes that were modified with AuNPs and functionalised with anti-FB1 and anti-DON antibodies and a Ag/AgCl pseudo-reference electrode etched on the ITO-coated glass and was integrated with a microfluidic channel. The binding of the toxin present in the sample to the antibody immobilized on the working electrode produced an electrochemical signal, which was detected by DPV. With this immunosensor set-up, a LOD of 97 pg/mL and 35 pg/mL could be achieved, and linear ranges of detection were 0.3–140 ng/mL and 0.2–60 ng/mL for FB1 and DON, respectively.

The statistical parameters of the measurements, the cross reactivity, and the matrix analysed of electrochemical immunosensors for DON and ZON detection are summarized in Table 2.

### 2.3. Piezoelectric Immunosensors

**Figure 4:**
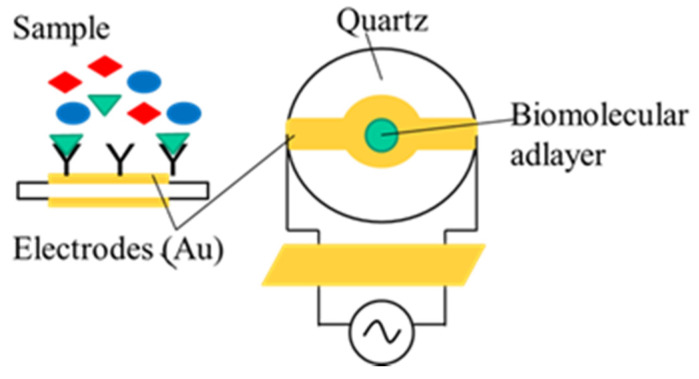
Structure principle of piezoelectric immunosensors.

Quartz crystal microbalance (QCM) is a piezoelectric effect-based mass measuring system. The QCM sensor is made of a quartz crystal disk cut to a specific orientation with respect to the crystal axes and sandwiched between two metal electrodes (usually gold) that can be made to oscillate at a defined frequency by applying alternating voltage. Its resonant frequency depends on the thickness of the crystal (Figure 4). The thinner the applied crystal, the higher its resonant frequency and sensitivity. QCM monitors the mass or thickness of the adlayers on the surface of the quartz crystal. The main advantages of QCM are high sensitivity, high stability, fast response, and low cost. It also provides label-free detection capabilities for biosensor applications. However, QCM faces some disadvantages, as its performance significantly depends on the temperature and other environmental parameters, and its sensitivity falls short of the requirements when measuring low molecular weight substances [64]. In order to fulfill the requirements of high sensitivity regarding mycotoxin detection (as they are low molecular weight compounds, so they cannot generate sufficient frequency changes) piezoelectric biosensors need to apply competitive inhibition immunoassay formats, or the signal has to be amplified by applying secondary antibodies or nanoparticles.

Although there are several examples of piezoelectric immunosensors for mycotoxin determination in the recent scientific literature [1,65,66,67,68,69], there have been very few developments for the piezoelectric determination of ZON and DON. Very recently a portable, label-free QCM immunosensor was introduced by Liu et al. [70] for ZON determination in different food matrices. In the sensor, ZON-ovalbumin conjugate was immobilised with EDC/NHS on the surface of the mercaptodecylic acid-modified chip. The frequency response caused by the specific binding of anti-ZON antibody (100 µg/mL) on the chip surface was detected in the presence or absence of ZON. A high sensitivity of ZON determination with a LOD as low as 0.37 ng/mL was obtained, with excellent selectivity and stability. The effectiveness of the sensor was verified in spiked corn, wheat flour, soy sauce, and milk samples, and satisfactory recoveries were attained. The sensor could be reused six times without any significant attenuation of frequency of the sensor chip (below 10%) and could be stored for fifteen days without significant signal loss. The sensor allowed quick ZON determination since it took five minutes to measure a sample.

Nolan et al. [71] developed a mass-sensitive microarray biosensor working under the same principle as QCM for multiplex mycotoxin determination. The sensor consisted of 4x16 mass-sensitive transducer pixels. Each pixel consisted of a zinc oxide piezoelectric layer sandwiched between two electrodes where the top electrode was coated with silicon dioxide with a thin gold layer on the top where mycotoxin conjugates were immobilized, and the entire set-up was mounted on the top of an acoustic mirror. With the optimised sensor, simultaneous determination of T2-toxin, ZON, and FB1 were examined. To assess sensitivity, IC_50_ values were calculated. Sensitivity of the multiplex assay were 6.1 ng/mL, 3.6 ng/mL, and 2.4 ng/mL, and the working range of the assay for T2, FB1, and ZON were 1.5–24.4 ng/mL, 0.9–14.3 ng/mL, and 0.6–9.6 ng/mL, respectively.

## 3. Sensors Based on Artificial Recognition Elements

MIPs are synthetic polymers that can be used to form an artificial receptor for the target analyte. They are synthesised by polymerisation of a monomer with a cross-linking agent in the presence of the target analyte. Upon cross-linking, a cavity is formed around the template, and after its removal, a recognition site appears for the target analyte. The formed polymer can be used as a recognition element in affinity-based sensors. MIPs are cheaper, have higher reusability, and are more resistant to pH and to ionic strength compared to antibodies; therefore, their use in sensor development is beneficial [72,73]. Aptamers are single-stranded nucleic acid (DNA or RNA) molecules with a high affinity to the target molecule. They are fabricated by an in vitro selection and amplification technology (SELEX) [74]. During several selection rounds, only those oligonucleotides are selected and enriched from the huge oligonucleotide library, which can bind with very high affinity to the specific molecular target. It can be stated that the affinity of aptamers can be as good as those of antibodies and in some cases, even better. In addition to that, aptamers are more stable and flexible and can be chemically modified, allowing their immobilisation in sensors.

### 3.1. Aptasensors

The use of aptamers over antibodies has been an emerging trend in the field of biosensor development in the last decades. Aptamers are synthetic, short, single-stranded nucleic acids with a high affinity to the target molecule. Due to their small size, high affinity, high stability, and specificity, they offer many advantages over conventional antibodies as recognition elements. Having such high affinity, aptamer-based homogeneous and heterogeneous sensors have emerged as a promising tool among the biosensors (Figure 3D). Fluorescent, colorimetric, and electrochemical detection methods are commonly used in these sensor systems. A fluorometric aptamer-based method was developed for simultaneous determination of ZON and FB1 using gold nanorods (AuNRs) and upconversion nanoparticles (UCNPs) [75]. In the sensor, UCNPs were modified with aptamers for ZON and FB1. The functionalised UCNPs were attached with their corresponding complementary nucleic acid (cDNA) sequences. To the AuNPs, different cDNAs for ZON and FB1 were attached, and the AuNPs and the UCNPs were assembled together. In the presence of ZON and FB1 in the sample, the biocomplex of UCNPs-AuNRs will be unstable, and the UCNP part separates from the complex, resulting in the recovery of fluorescence signals. Under 980-nm laser excitation, ZON was detected at 606 nm and FB1 at 753 nm. The LODs of the assay for ZON and FB1 were 1 pg/mL and 3 fg/mL, respectively, with average recoveries from spiked maize samples of 90 to 107%.

Similarly, a fluorescent aptasensor created through UCNPs was presented for ZON determination in corn and beer [76]. A ZON-specific aptamer was used as a recognition probe, while the complementary strand was adopted as a signal probe. In the sensor, ZON aptamer was immobilised on the surface of the amino-modified magnetic nanoparticles, while cDNA was immobilised on the surface of UCNPs and were mixed together to form the duplex structure. When ZON is present in the sample, the ZON-aptamer dissociates from the complex and binds to ZON; therefore, a decrease in the fluorescence intensity occurs. For excitation, a 980 nm laser light was used, and ZON was detected at 543 nm. In this sensing platform, a linear response of 0.05–100 ng/mL was obtained between the fluorescence signal and ZON levels with a LOD of 0.126 μg/kg in corn and 0.007 ng/mL for beer, demonstrating that the developed aptasensor offered a novel approach for ZON analysis in food. Li et al. [77] presented an aptasensor for ZON determination in maize samples that was based on fluorescence resonance energy transfer (FRET) between fluorescent UCNPs modified with aptamer as donors and graphene oxide modified with carboxyl groups as acceptor. When UCNPs and functionalised graphene oxide were at a close distance (less than 10 nm), fluorescence quenching was noticed. As the aptamers prefer to bind to their corresponding mycotoxins, in the presence of ZON, the formation of aptamers change, so aptamer modified-UCNPs are far away from the surface of the functionalised graphene oxide. The presented sensor had a wide working range (0.005–100 ng/mL), good stability (28 days), and the results showed that the aptamer-UCNP-functionalised graphene oxide probe provided a rapid, accurate, and simple to use system for ZON detection.

Azri et al. [78] fabricated an electrochemical label-free competitive aptasensor for ZON determination. The sensor had a working range of 0.01 to 1000 ng/mL ZON concentration with a LOD of 0.017 ng/mL. With the established aptasensor, ZON concentrations of maize grain extracts were determined. For ZON determination, He et al. [79] described a voltammetric aptasensor based on the use of porous platinum nanotubes/AuNPs and thionine-labelled graphene oxide for signal amplification. The working range of the aptasensor was 0.5 pg/mL to 0.5 μg/mL for ZON with a LOD of 0.17 pg/mL.

Recently, an aptasensor for ultrasensitive detection of ZON by using CoSe_2_ nanocrystal /AuNRs, 3D structured DNA-PtNi@Co-metal-organic framework networks, and nicking enzyme as signal amplification system was proposed [80]. In the sensor DPV detection method was used for ZON determination. Comparing to other ZON methods, the aptasensor possessed outstanding sensitivity (LOD = 1.37 fg/mL) and wider linear range (10.0 fg/mL to 10.0 ng/mL). In addition, no additional substrate was needed compared to conventional enzymatic amplification by substrate cycling. Ong et al. [81] described a novel aptasensor for DON determination where they used iron nanoflorets graphene nickel (INFGN) as a transducer. The INFGN enabled a feasible bio-capturing due to its large surface area where the hydroxyl groups act as linkers. The biomolecular interaction in the sensor results in conductivity changes determined by current-voltage measurement using a picoammeter. The sensor showed good stability, it retained 30.65% of its activity after 48 h, and provided highly sensitive and selective detection of DON at a LOD of 2.11 pg/mL. Another research group used the 3D sakura-shaped copper (II) ions@L-glutamic acid nano-metal-organic coordination polymers (MOCPs) for the first time to develop an electrochemical aptasensor for ultrasensitive detection of ZON. Cronoamperometry was used for ZON determination. Under optimal conditions, dynamic range of 1 fg/mL to 100 ng/mL ZON was obtained with a LOD of 0.45 fg/mL [82].

Han et al. [83] presented a co-reduced molybdenum disulphide and gold nanoparticles (rMoS_2_-Au)-based electrochemical aptasensor for ZON and FB1 simultaneous detection. For sensor fabrication on the surface of the reduced molybdenum disulphide and AuNPs, coated glassy carbon electrode ZON and FB1 aptamers were conjugated. The corresponding cDNA sequences and thionine and 6-(ferrocenyl)hexanethiol as probes for ZON and FB1 detection were immobilised on AuNPs, which were bound to the aptamers through the complementary base pairing. In the presence of ZON and FB1, the labelled corresponding cDNAs are replaced by the target molecule, resulting in signals proportional to the concentrations of the analytes. Differential pulse voltammetry was used to detect the concentrations of the mycotoxins. The aptasensor allowed ZON and FB1 determination in the range of 1×10^−3^–10 ng/mL and 1×10^−3^–1×10^2^ ng/mL, respectively. The sensor possesses the LOD of 5× 10^−4^ ng/mL. The performance of the aptasensor was successfully demonstrated in real maize samples with satisfactory recoveries.

The statistical parameters of the measurements, the cross reactivity, and the matrix analysed of aptasensors for DON and ZON detection are summarized in Table 3.

### 3.2. Molecularly Imprinted Polymer Sensors

In recent years, MIPs are widely used primarily in the SPR biosensor technique. Comparing to antibodies, MIPs are more resistant to harsh regeneration conditions and are less likely to lose their binding capability. Although there are several methods to prepare MIPs for sensor applications, the most common method is the in situ polymerisation directly onto the sensor surface (Figure 3C). Choi et al. [84] developed an SPR sensor for ZON determination using MIPs as recognition elements. On the gold sensor surface, a molecularly imprinted polypyrrole film was prepared by electropolymerisation in the presence of ZON as a template. The sensor had a linear response in the range of 0.3–3000 ng/mL for ZON, and the LOD was 0.3 ng/g in corn samples. They also prepared a similar MIP-based SPR sensor for the determination of DON in which the linear measuring range was between 0.1–100 ng/mL. The selectivity of the MIP layer for 3- and 15-acetyl-DON was found to be 19% and 44%, respectively [85].

Sergeyeva et al. [86] developed a novel sensor for ZON detection in cereals suitable for field application. A ZON-selective urethane-acrylate MIP membrane was used to form the sensor, and the natural fluorescence of ZON was analysed by a Spotxel^®^Reader smartphone application. In the direct sensing mode, the LOD of ZON was 126 μg/kg, but the competitive sensing mode allowed a sensitivity improvement to a LOD of 1.26 μg/kg.

## 4. Conclusions

Quick, easy to use, and sensitive determination of mycotoxins are extremely important in the food and feed industry because the use of mycotoxin-contaminated commodities poses health risks to the consumers and to livestock as well. The application of biosensors could be an expedient alternative over advanced instrumental chromatographic techniques, as they offer cost-effective, rapid, portable, on-site determination possibilities of mycotoxins. Although developments of several immunosensors for mycotoxin determination have been reported in the scientific literature, they are mainly focused on aflatoxin and ochratoxin as target analytes, but much less attention has been paid to the determination of ZON and DON, and the reports dealing with masked mycotoxins are unduly rare. For the detection of small molecular mass analytes, substantial advances have occurred in the fields of electrochemical and optical immunosensing. Efforts for both types of these sensors are aimed to improve biosensor characteristics, including sensitivity, selectivity, fast response, and low cost; therefore, incorporation of nanomaterials (nanoparticles, nanorods, nanotubes, nanowires) into biosensors are being widely studied. The advantages of using nanoparticles are that they either increase the sensor surface area suitable for biomolecule immobilisation or enhance the signal derived from the immunocomplex formation. It has been found that nanomaterials applied in biosensors as signal amplification tags can improve sensitivity and can reduce the LOD by several orders of magnitude. The use of the favourable properties of nanomaterials in the determination of mycotoxins via immunosensors is particularly important, as these analytes are low molecular weight substances; therefore, their detection is challenging. Another emerging trend in biosensor development is the application of aptamers and MIPs as synthetic receptors in biosensor fabrication. During the past decade, the focus of the attention has turned towards the development of aptasensors due to the stability, selectivity, and sensitivity of these oligonucleotide-type artificial recognition elements. Despite new achievements, areas demanding more research still exist, particularly in the fields of masked mycotoxins and multiplex mycotoxin determination.

## Figures and Tables

**Table 1:** Statistics of measuring parameters, cross reactivity, and the matrix analysed of optical immunosensors for DON and ZON detection.

Mycotoxin	Method	Detection Range	LOD	Matrix	Selectivity/cross Reactivity	Reference
**AFB1** **OTA** **ZON** **DON**	SPR	0.99–21.92 ng/mL 1.98–28.22 ng/mL 10.37–103.31 ng/mL 5.31–99.37 ng/mL	0.59 ng/mL, 1.27 ng/mL, 7.07 ng/mL 3.26 ng/mL	Spiked corn and wheat	AFB2 19.1% OTB 6.2% α-ZEL 15,3% 15-AcDON 16.2%	[34]
**DON** **ZON** **T-2**	iSPR	48–2827 µg/kg 54–790 µg/kg 42–1836 µg/kg	15 µg/kg 24 µg/kg 12 µg/kg	wheat	15-AcDON 150% α-ZEL 104% HT-2 n.s.	[36]
**T-2** **T2-G**	iSPR		1.2 ng/mL 0.9 ng/mL	spiked wheat	15-AcDON < 1% HT-2Glc < 1% HT-2 < 1%	[37]
**AFB1** **OTA** **ZON**	iSPR		8 pg/mL 30 pg/mL 15 pg/mL	spiked peanut	n.d.	[38]
**DON**	OWLS	0.01–100 ng/mL	0.005 ng/mL	spiked wheat flour	n.d.	[40]
**ZON**	OWLS	0.01–1 pg/mL	0.002 pg/mL	spiked maize	α -ZEL 25.2% Zeranol 12.8%	[41]
**ZON**	PW	0.01–1000 ng/mL	0.01 ng/mL	ZON standard	AFB1 n.s. OTA n.s.	[42]
**DON**	WLRS	62.5 μg/kg–12.5 mg/kg	62.5 μg/kg	spiked maize wheat	3-AcDON 929% 3DON-Glc 23%	[44]
**DON**	DON-Chip	0.01–20 μg/g	4.7 ng/g	food, feed	n.d.	[45]
**ZON** **DON**	NIR-based LFIA	0.012–0.33 ng/mL 0.082–6.7 ng/mL	0.55 μg/kg 3.8 μg/kg	maize	AFB1 <1% FB1 <1% OTA <1% T-2 <1%	[46]
**DON** **OTA** **AFB1**	Microfluidic immunoassay		10 ng/mL 40 ng/mL 0.1 ng/mL	spiked corn feed	OTA, AFB1 n.s. DON, AFB1 n.s. OTA, DON n.s.	[47]
**FB1** **ZEN** **T-2** **DON** **AFB1**	LFIA	0.5–10 μg/kg 0.25–5 μg/kg 0.3–1 μg/kg 1–20 μg/kg 0.25–0.5 μg/kg	10 μg/kg 2.5 μg/kg 1.0 μg/kg 10 μg/kg 0.5 μg/kg	maize	α-ZEL 70.6% Zeranol 32% HT-2 37% 3-AcDON 347% 15-AcDON 34% AFM1 45%	[48]

Deoxynivalenol (DON), 15-acetyl-deoxynivalenol (15-AcDON), 3-acetyl-deoxynivalenol (3-AcDON), Deoxynivalenol 3-glucoside (3DON-Glc), Zearalenone (ZON), α-zearalenol (α-ZEL), β-zearalenol (β-ZEL) α-zearalanol (Zeranol), Ochratoxin A (OTA), Ochratoxin B (OTB), Aflatoxin B1 (AFB1), Aflatoxin B2 (AFB2), Aflatoxin M1 (AFM1), HT-2-glucoside (HT-2Glc), Fumonisin B1 (FB1), Fumonisin B2 (FB2), T-2 glucoside (T2-G), signal is not significant (n.s.), no data (n.d.), surface plasmon resonance (SPR), Imaging surface plasmon resonance (iSPR), optical waveguide lightmode spectroscopy (OWLS), planar waveguide (PW), white light reflectance spectroscopy (WLRS), near-infrared fluorescence-based lateral flow immunosensor (NIR-based LFIA).

**Table 2:** Statistics of measuring parameters, cross reactivity, and the matrix analysed of electrochemical immunosensors for DON and ZON detection.

Mycotoxin	Method	Detection Range	LOD	Matrix	Selectivity	Reference
ZON	Amperometry	0.1 to 100 pg/mL	0.15 pg/mL	spiked maize	n.d.	[60]
ZON	DPV	0.25–256 ng/mL	0.25 ng/mL	spiked beer, wine	AFB1 AFM1 85–90% OTA OTB	[61]
ZON	Amperometry	1.88–45 ng/mL	0.57 ng/mL	*Amaranthus cruentus* seeds	n.d.	[62]
FB1 DON	DPV	0.3–140 ng/mL 0.2–60 ng/mL	97 pg/mL 35 pg/mL	spiked corn sample	n.d.	[63]

Deoxynivalenol (DON), Zearalenone (ZON), Fumonisin B1 (FB1), Aflatoxin B1 (AFB1), Aflatoxin M1 (AFM1), Ochratoxin A (OTA), Ochratoxin B (OTB), differential pulse voltammetry (DPV), no data (n.d.)

**Table 3:** Statistics of measuring parameters, cross reactivity, and the matrix analysed of aptasensors for DON and ZON detection.

Mycotoxin	Method	Detection Range	LOD	Matrix	Selectivity	Reference
ZON FB1	Fluorometric method	0.05–100 μg/L 0.01–100 ng/L	0.01 μg/L 0.003 ng/L	spiked corn sample	AFB1, OTA, PAT, OTB n.s.	[75]
ZON	Upconversion fluorescence	0.005–100 ng/mL	0.0018 ng/mL	maize	AFB1, AFB2, OTA, DON, FB1 ≈Low n.d.	[77]
ZON	Fluorescense	0.05–100 μg/L	0.126 μg/kg	spiked corn	AFB1, AFB2, OTA, FB1, FB2, a-ZEL, β-ZEL <13%	[76]
ZON	Square wave voltammetry	0.01–1000 ng/mL	0.017 ng/mL	spiked maize	α-ZEL, β-ZEL, ZON-14-Glc, DON, FB1 ≈high n.d.	[78]
ZON	Voltammetry	0.5 pg/mL–0.5 μg/mL	0.17 pg/mL	spiked maize	DON, AFB1, PAT ≈Low n.d.	[79]
ZON	DPV	10.0 fg/mL– 10.0 ng/mL	1.37 fg/mL	spiked maize	DON, OTA, AFB1, PAT, FB1 n.s.	[80]
DON	Voltammerty	1 pg/mL–1 ng/mL	2.11 pg/mL	spiked rice	OTA, ZON <14%	[81]
ZON	Cronoamperometry	1 fg/mL to 100 ng/mL	0.45 fg/mL	spiked beer	T-2, OTA, FB1, AFB1 n.d.	[82]
ZON FB1	DPV	0.001–10 ng/mL 0.001–100 ng/mL	0.0005 ng/mL	maize	α-ZEL, FB2, AFB1, DON, T-2, OTA n.d.	[83]

Deoxynivalenol (DON), Zearalenone (ZON), α-zearalenol (α-ZEL), β-zearalenol (β-ZEL), Zearalenone-14-Glucoside (ZON-14-Glc), Ochratoxin A (OTA), Ochratoxin B (OTB), Aflatoxin B1 (AFB1), Aflatoxin B2 (AFB2), Aflatoxin M1 (AFM1), Fumonisin B1 (FB1), Fumonisin B2 (FB2), Patulin (PAT), differential pulse voltammetry (DPV), signal is not significant (n.s.), no data (n.d.).
